# Estimation of the flow motion in SSFP images using moving shadows; reliable or not?

**DOI:** 10.1186/1532-429X-17-S1-P75

**Published:** 2015-02-03

**Authors:** Saeid Ahmadinia, Abbas N Moghaddam

**Affiliations:** 1 BME, Tehran Polytechnic, Tehran, Iran, the Islamic Republic of; 2 Radiology, UCLA, Los Angeles, CA, USA

## Background

SSFP plays an important role in cardiovascular MRI due to its high speed and unrivaled contrast between blood and myocardium. Some "flow artifacts", however, rises because blood flow prevents spins from reaching the steady state. One artifact in this category is the moving shadows in the blood pool that appear to represent the actual flow behavior. In this study, we show that the shadows are not necessarily representing actual flow behavior. Therefore, cardiologists should consider the moving shadows cautiously to avoid misinterpretations.

## Methods

**Figure 1:**
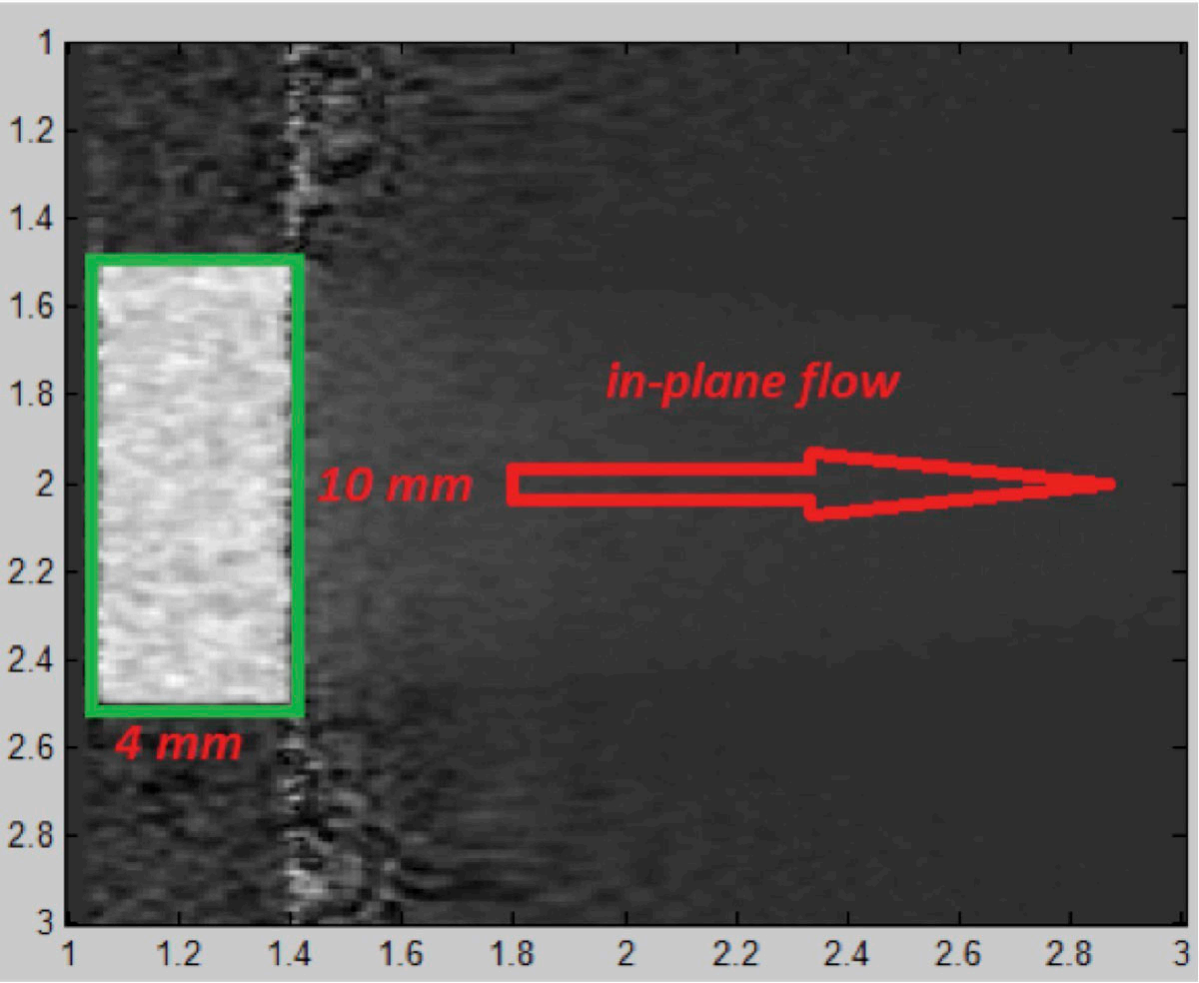
**The geometry of the simulated flow:** The through plane flow is shown in the bright rectangle in the left. The in-plane flow is also indicated.

A simulator has been developed based on Bloch equation. After each excitation, it repeatedly updates the position and magnetization of each moving or stationary spin in the slice. An oscillating flow, with a full-wave rectified sinusoidal form, was picked up for the simulation. The in-plane left to right flow has a parabolic profile with a period of 1 second. Additionally, fresh spins enter to the slice by a through-plane flow in the left side of the plane, resembling the effect of a valve, stenosis or anything that takes spins in and out of the excitation slice. The phase encoding direction is perpendicular to the direction of in-plane flow (fig. 1).

## Results

**Figure 2:**
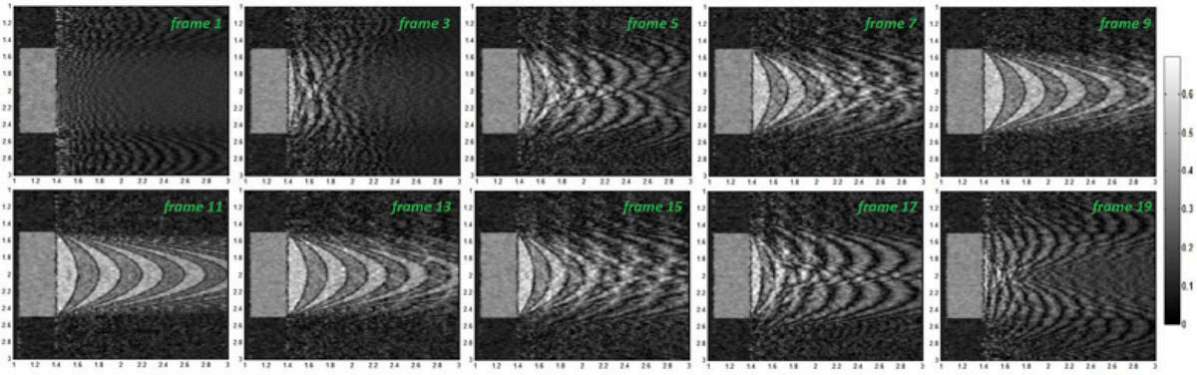
**Every other reconstructed frame among the total number of 20 frames:** In-plane flow has a full-wave rectified sinusoidal form with a maximum velocity of 40 cm/s.

The simulation was performed for in-plane velocity peak of 40 cm/s. Fig. 2 illustrates 10 frames of the simulation while 20 frames has been acquired totally. The parabolic profile of in-plane flow is visible in the frames moving to right and left.

As figure 2 illustrates, shadows move toward right proportional to the in-plane velocity (figure 2 frames 1, 3, 5, 7, 9) for half of the period and after reaching the peak velocity, while the reference flow is still moving toward right, they seem to return to left (compare frames 11 and 13). This can result in some improper judgments about the direction of the flow.

## Conclusions

In cardiac imaging with SSFP sequence, flow artifacts appear in images since fluid motion prevents spins to reach steady state. One should note that flow artifacts showing themselves as moving shadows, are not always reliable for considering actual flow behavior. We showed the shadows motion could be in the opposite direction of actual flow.

## Funding

N/A.

